# The action of aminoguanidine on the liver of trained diabetic rats

**DOI:** 10.1186/2251-6581-12-40

**Published:** 2013-07-09

**Authors:** Edmara Tereza Meira e Nico, Patrícia Rosa de Oliveira, Leonardo Peres de Souza, Franco Dani Campos Pereira, Maria Andréia Delbin, Angelina Zanesco, Maria Izabel Camargo-Mathias

**Affiliations:** UNESP-University Estadual Paulista, Avenida 24 A, 1515, 13506-900, Rio Claro, SP CP 199 Brazil

**Keywords:** Aminoguanidine, Diabetes type 1, Rats, Liver, Physical exercises

## Abstract

**Background:**

This study evaluated the effect of aminoguanidine on liver of diabetic rats subject to physical exercises using histological and histochemical techniques.

**Methods:**

The rats used in this study were divided into five groups: sedentary control, sedentary diabetic, trained diabetic, sedentary diabetic and treated with aminoguanidine, trained diabetic and treated with aminoguanidine.

**Results:**

The results showed no effect of aminoguanidine on the liver tissue, although there was improvement with exercise training showing cytological, morpho-histological and histochemical alterations in liver cells of animals from groups trained diabetic and/or treated diabetic compared to those individuals in the sedentary control and sedentary diabetic. These changes included: hepatocytes hypertrophy, presence and distribution of polysaccharides in the hepatocytes cytoplasm and, especially, congestion of the liver blood vessels.

**Conclusion:**

Our results suggest that aminoguanidine is not hepatotoxic, when used at dosage of 1 g/L for the treatment of diabetes complications, and confirmed that the practice of moderate physical exercise assuaged the damage caused by diabetes without the use of insulin.

**Electronic supplementary material:**

The online version of this article (doi:10.1186/2251-6581-12-40) contains supplementary material, which is available to authorized users.

## Introduction

Diabetes mellitus (DM) is a metabolic disorder characterized by lack or deficiency of insulin secretion or even by the peripheral resistance to it [1]. The insulin deficiency affects the metabolism of carbohydrates, lipids and proteins resulting in hyperglycemia and glycosuria, responsible for degenerative diseases of arterial system, eyes and the brain [1]. In fact, it was considered the fifth major cause of death in the world [2].

Regular physical activity induces structural and functional changes which increase the metabolism of the organism [3]. During physical activity several sources of energy are used, including glycogen from muscles and liver, as well as the triglycerides stored in the adipose tissue. The main source of energy used during the first 20 or 30 minutes of physical exercise of moderate intensity is the glycogen. After this period, the oxidation of lipids is predominant [3, 4].

The physical exercise improves catchment of glucose by the cells and increases the glycogenesis, promoting, at the same time, adaptations in the skeletal muscle which stimulate the increase of the utilization of lipids [3]. Physical exercises of moderate intensity would be recommended to improve the metabolic control of diabetics, particularly those who are resistant to insulin, except when there are contraindications [5].

The practice of physical exercises brings visible results, such as the decrease in the insulin secretion and increase in glucagon secretion, due to the activity of the sympathetic nervous system, which directly innerves the Langerhans islets (endocrine portion of the pancreas), via alpha adrenergic receptors (predominantly) [6].

Thus, the physical exercise would be one of the responsible factors for the decrease in insulin secretion and increase in glucagone secretion, exceeding the even the same metabolic stimuli as glycemia, which would either not be altered or could be elevated during prolonged exercise [6].

Data from literature demonstrate the occurrence of the beneficial effects of physical training on DM, either by the increase of sensibility to insulin or by the effect on its secretion [7].

The process of insulin secretion by pancreatic cells is modulated by several factors including nutrients, neurotransmitters and hormones. The main stimulating factor for the insulin secretion is the glucose, the only nutrient-*in vitro*-which promotes (alone) the release of insulin [8].

The most important effect of insulin in the organism is to promote the transportation of glucose to the interior of the cells, in special muscular cells, adipose and hepatic [8]. The deficiency of insulin can cause, among other consequences, the decrease capability of synthetizing and accumulating glycogen in the liver [6].

Insulin is a peptide hormone secreted by pancreas, such hormone favors the storage of substrates and the maintenance of the tissues. In normal levels of glycemia (between 75 and 100 mg/mL) and in the absence of insulin, the nervous tissue would be the only to use the circulating glucose in amounts sufficient to the maintenance of its metabolic needs. The presence of insulin would then be necessary to ensure the entrance of adequate amounts of glucose in other cells. Independently of the cause, the lack of insulin would alter the metabolism of the glycides, lipids and proteins in the organisms [9].

The occurrence of vascular damages in diabetic individuals would be related to the formation of AGEs (advanced glycation end products), molecules formed from the glycation (aminocarbonyl interactions–non-enzymatic nature) of proteins and lipids consequent from hyperglycemia which can attach to the walls of sanguineous vases [10]. In this sense it is known that aminoguanidine would be an anti-AGEs agent, being efficient in the therapy against the formation of atherosclerosis plaques in vases of diabetic rats [11].

Aminoguanidine is an anti-AGE which prevents the formation of ROS (Reactive Oxygen Species) and lipid peroxidation in cells which has been shown to be effective in the therapy against the formation of atherosclerotic plaques in blood vessels in diabetic rats [11, 12]. By binding to the reactive carbonyl groups Amadori products formed during the Maillard reaction, avoids thus the formation of AGEs [13].

Aminoguanidine prevents the formation of AGEs due to the presence of hydrazine in its chemical structure, which reacts with glyoxal, methylglyoxal and 3-deoxyglucosone [14]. In addition to the effects on advanced glycation, the aminoguanidine acts in a specific way inhibiting the activity of the enzyme iNOS (nitric oxide synthase) reducing the nitrosative stress [15].

The liver is a gland, attached to the digestive system, responsible for the storage and the metabolism of the nutrients absorbed by the intestines, such as lipids, proteins and carbohydrates, and also for the collection and transformation of metabolites from several parts of the organism [16]. According to Hinton and Laurén [17], the liver is also the organ responsible for the detoxification of xenobiotic compounds, being essential for their metabolism and excretion.

Considering the information exposed above, this study aimed to analyze, using cytological, histological and histochemical techniques, fragments of diabetic rats’ livers subjected to treatment with aminoguanidine and physical exercises, detecting the potential of the action of this substance and possible damages that this chemical would cause to the individuals treated with medications containing it in their composition.

## Material and methods

### Animals

Twenty five male albino rats (Wistar) obtained from the Central Biotery of Universidade Estadual Paulista (Sao Paulo State University)–UNESP, Campus of Botucatu, SP, Brazil and weighing between 180 and 200 g, were used. During the whole experiment, the animals were maintained in collective plastic boxes (three animals/box) in the Maintenance Biotery of the Physical Education Department of the Bioscience Institute, UNESP, campus of Rio Claro, SP, Brazil, under controlled conditions (22°C ± 2°C, 50% of humidity and photo period of 12 h) and received water and commercial food Purina (Campinas, SP, Brazil) *ad libitum*.

### Induction to diabetes

The animals induced to diabetes type 1 fasted for 18 hours and after this period received a single dose (intraperitoneal) of estreptozotocine (Sigma-Aldrish CO, Saint Louis, MO, EUA) at the concentration of 60 mg/kg diluted citrate buffer 0.1 M and pH 4.5 [18].

### Obtainment of pimagedine (aminoguanidine)

The aminoguanidine (AG) (Sigma-Aldrish CO, Saint Louis, MO, EUA) was administered via drinking water in solution of 0.1% (1 g/L) of AG for 8 weeks after the induction of diabetes [11].

### Experimental model

The rats to be studied were divided into five groups: sedentary control (C/SD), sedentary diabetic type 1 (DB/SD), trained diabetic type 1 (DB/TR), sedentary diabetic type 1 and treated with aminoguanidine (DB/SD-AG), trained diabetic type 1 and treated with aminoguanidine (DB/TR-AG).

### Physical exercise program

The chosen physical training was running on treadmill. In the first week, the animals were subjected to a period of adaptation to the treadmill and to the speed which was progressively increased ranging from 5 (on the first day) to 10 m/min (on the fifth day). The speed and the duration of the sessions increased progressively until the animals could remain running for 60 minutes. Only animals adapted to this activity were chosen and used in this study.

In the second week the physical training started with the speed of 10 m/min, increasing progressively according to the group of animals until reaching the final speed correspondent to the maximal lactate steady state (MLSS) anteriorly established for rats. A group of diabetic rats was specially induced for the determination of the training’s intensity. The intensity of the treadmill training for diabetic type 1 animals was determined through MLSS test. It is defined as the highest intensity of activity that can be maintained without the continuous accumulation of blood lactate, where the aerobic metabolism predominates over the anaerobic, being an important marker of the aerobic capacity [19, 20]. The blood lactate is an indicator of metabolism transition and authors consider the concentration of 4,0 mmol/L as a marker of the start of lactate accumulation [21, 22]. The animals presented stabilization in the lactate concentration at speeds 10 m/min (3,3 ± 0,3 mmol/L) and 15 m/min (3,6 ± 0,3 mmol/L). However, increasing accumulation of lactate was observed at 20 m/min (6,0 ± 1,1 mmol/L). Considering the definition of MLSS we used the speed of 15 m/min for the physical training, 5 days/week, 60 minutes each session, for 8 weeks, during the morning. At the end of the total period of training, the 12-hour fasting animals rested for 48 hours. Then the animals were anesthetized with 30 mg/Kg of intraperitoneal sodium thiopental and a longitudinal incision was made in the abdomen for the collection of arterial blood sample (7 mL), obtained from the descending branch of the aorta. The animals were then sacrificed by exsanguination following anesthesia. This study was approved by the Ethics Committee of Animal Experimentation CEEA/UNICAMP Biology Institute (IB) (protocol number 1753–1).

## Methods

### Histology

After being sacrificed, the rats were placed on wax plates for dissection. Fragments of the liver were removed with the use of dissecting scissors, tweezers and absorbent paper. Next, they were fixed for 24 hours in paraformaldehyde at 4% (or other described by the technique), transferred to phosphate buffer 10% (0.1 M pH 7.4) for more 24 hours. After they were dehydrated in crescent ethanol solutions at 70, 80, 90 and 95% for 30 minutes each bath, embedded in Leica resin for 24 hours and transferred to plastic molds which were posteriorly filled with polymerized Leica resin. After the polymerization of the resin, all the blocks containing the material were sectioned at 3 μm and stained with hematoxylin and eosin. The material was examined under photomicroscope Motic BA300.

### Histochemistry

Aiming to detect the presence, frequency and distribution of polysaccharides and protein components in the livers of the rats here studied, after the euthanasia fragments of the livers were collected and subjected to the following histochemical techniques:

#### Bromophenol blue technique for the detection of proteins (according to Pearse [23])

The fragments were fixed in paraformaldehyde at 4% for 24 hours. The sections were stained with Bromophenol blue for two hours in ambient temperature. After washed in acetic acid at 0.5% for 5 minutes and running water for 15 minutes, the sections were quickly passed in tertiary butyl alcohol. Next, they were air-dried, diaphanized and mounted in Canada balsam.

#### PAS technique for the detection of polysaccharides (according to Junqueira and Junqueira [24])

Fragments of the liver were fixed in aqueous Bouin for 24 hours. The slides containing the sections remained for 10 minutes in periodic acid solution at 0.4%, quickly washed in distilled water and subjected to Schiff reagent for 1 h, in the dark. Posteriorly, three washes in sulfurous water were performed for 3 minutes each and then the slides were washed for 30 minutes in running water. After dried, the slides were diaphanized in xylol and mounted in Canada balsam.

The material was examined under MOTIC BA 300 photomicroscope, coupled with an INTEL microcomputer.

## Results

### Liver histology

The results obtained show the alterations in the hepatic cells obtained from rats induced to diabetes, subjected to physical exercises in treadmill and treatment with aminoguanidine.

#### Sedentary control group (C/SD)

**Figure 1:**
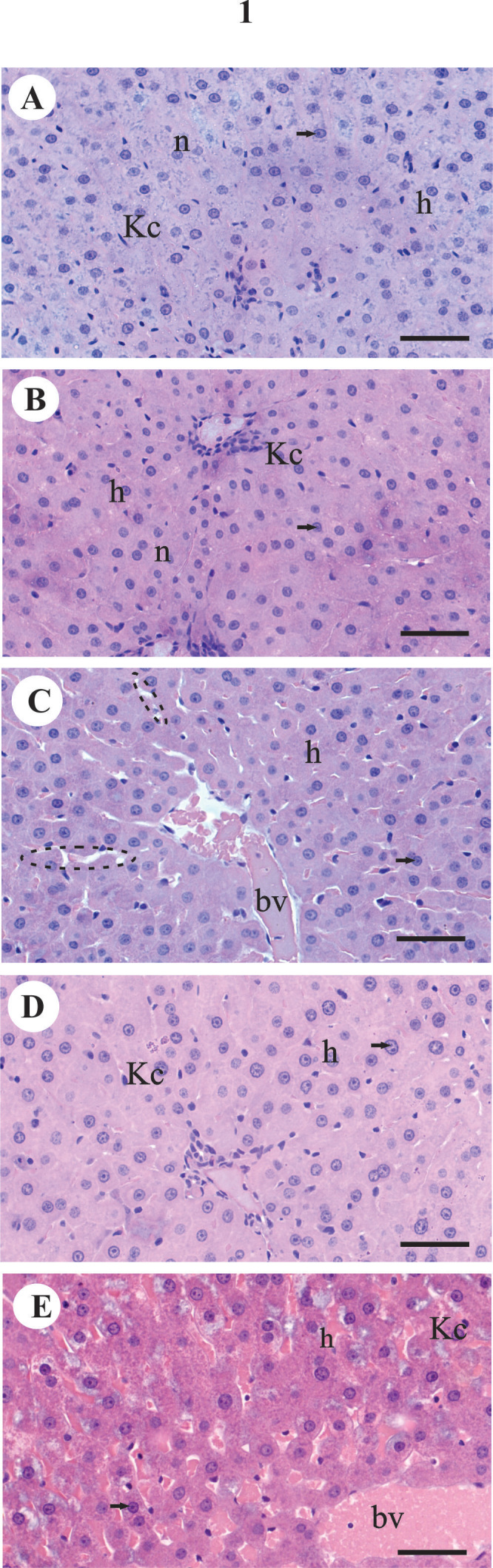
**Histological sections of the liver of rats. A-E.** Hematoxylin and eosin (HE) staining. **A.** Sedentary control (C/SD), **B.** Sedentary diabetic type 1 (DB/SD), **C.** Trained diabetic type 1 (DB/TR), **D.** Sedentary diabetic type 1 and treated with aminoguanidine (DB/SD-AG), **E.** Trained diabetic type 1 and treated with aminoguanidine (DB/TR-AG). c = hepatic sinusoidal capillaries, h = hepatocytes, Kc = Kupffer cells, n = nucleus of hepatocytes; bv = blood vessels, arrow = nucleoli, dotted line = interplate spaces. Scale bars: A-E = 0.02 mm.

That results revealed the liver of the individuals from C/SD group presents polygonal hepatocytes organized in extended anastomosed plaques. The cytoplasm of these cells is little acidophil, presenting reduced number of basic components (Figure 1A). The hepatocytes present one or two large nuclei, central, round-shaped and strongly stained by hematoxylin. In some hepatocytes it is still possible to observe the presence of one or more nucleoli (Figure 1A).

There are numerous hepatic sinusoidal capillaries between the hepatocytes plates, to which walls the Kupffer cells adhere, observed through light microscopy by the presence of their elongated and/or triangular and strongly stained nuclei (Figure 1A).

#### Sedentary diabetic type 1 (DB/SD)

The alterations found in the liver of the individuals from this group evidenced the morphophysiological modifications caused by diabetes. The hepatocytes are smaller, as well as their nuclei. The cytoplasm is more acidophil; i.e., with a great number of basic components (Figure 1B).

The nuclei of Kupffer cells present the same morphology observed in the individuals from the sedentary control group (Figure 1B).

#### Trained diabetic type 1 (DB/TR)

The individuals from this group, contrarily to what was observed in the previous groups, presented hypertrophied hepatocytes. Their nuclei is also larger and with evident nucleoli. The cytoplasm of the hepatocytes is homogeneous and acidophil (Figure 1C). Some interplate vacuolation is observed in these individuals, contrarily to the previous groups (Figure 1C).

In this group, the lumen of the sinusoidal capillaries is full of red blood cells (erythrocytes) (Figure 1C).

#### Sedentary diabetic type 1 and treated with aminoguanidine (DB/SD-AG)

The liver of the individuals (DB/SD-AG) shows hepatocytes, with increased size in relation to those found in the trained diabetic group (DB/TR). The nucleus of these cells is also larger and the cytoplasm presents high acidophili (Figure 1D).

A decrease in the presence of vacuolation is observed (intercellular spaces) contrarily to what was observed in the trained diabetic group (Figure 1D).

#### Trained diabetic type 1 and treated with aminoguanidine (DB/TR-AG)

The livers of the trained and treated individuals (DB/TR-AG) present significant histological alterations when compared to those from control group (Figure 1E).

In this group the hepatocytes are more hypertrophied, with shapes ranging from round to oval, consequence of the hypertrophy (Figure 1E).

The nuclei of the hepatocytes are large and nucleoli are evident. Acidophil granules are found in their cytoplasm. The presence of cytoplasmic vacuoles is verified in most hepatocytes. Some hepatocytes present great vacuolation, mainly around the nucleus (Figure 1E).

Great vacuolation is found between the hepatocytes plaques, as well as the presence of a great number of erythrocytes (Figure 1E).

There is a decrease in the number of Kupffer cells (observed by the presence of elongated and/or triangular); however, atypical, pyknotic and smaller nuclei are observed (Figure 1E).

### Liver histochemistry

#### Detection of proteins–bromophenol blue technique

##### Sedentary control group (C/SD)

**Figure 2:**
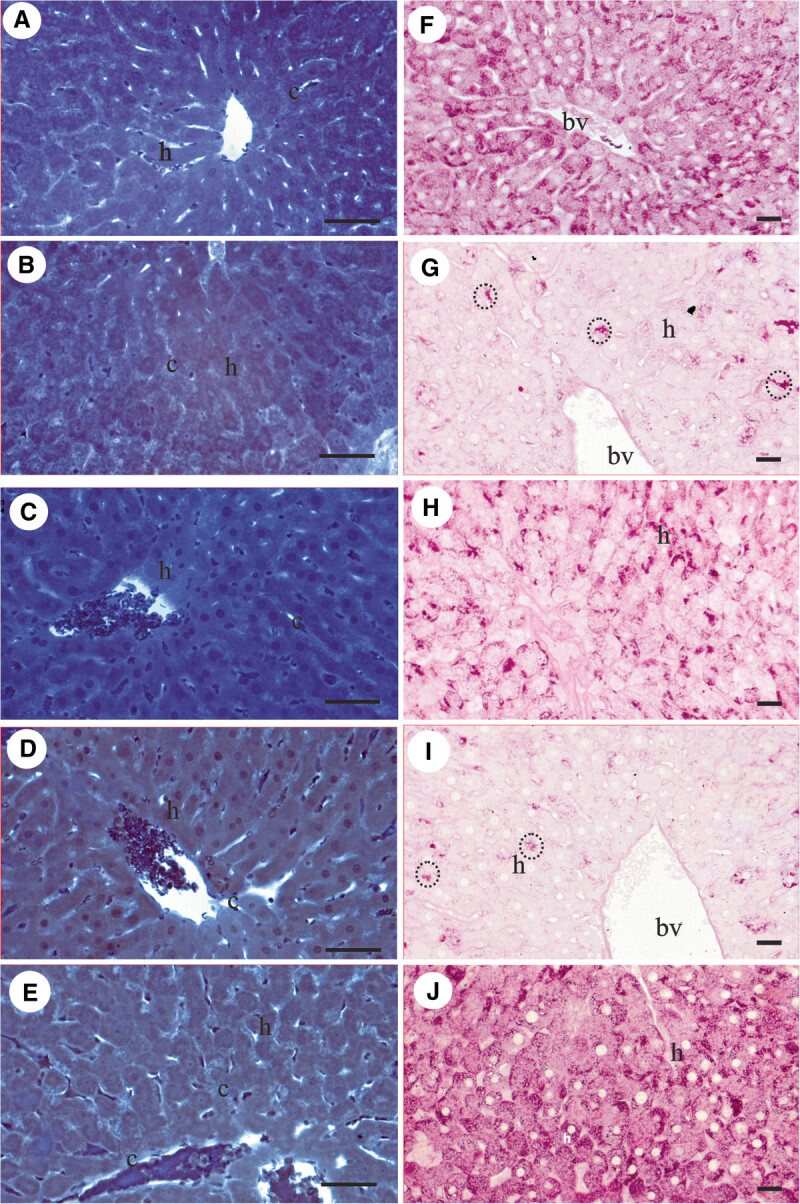
**Histological sections of the liver of rats. A-E.** Bromophenol blue staining to detect proteins. **A.** Sedentary control (C/SD), **B.** Sedentary diabetic type 1 (DB/SD), **C.** Trained diabetic type 1 (DB/TR), **D.** Sedentary diabetic type 1 and treated with aminoguanidine (DB/SD-AG), **E.** Trained diabetic type 1 and treated with aminoguanidine (DB/TR-AG). **F-J.** PAS staining to detect polysaccharides. **F.** Sedentary control (C/SD), **G.** Sedentary diabetic type 1 (DB/SD), **H.** Trained diabetic type 1 (DB/TR), **I.** Sedentary diabetic type 1 and treated with aminoguanidine (DB/SD-AG), **J.** Trained diabetic type 1 and treated with aminoguanidine (DB/TR-AG). c = hepatic sinusoidal capillaries, h = hepatocytes, bv = blood vessels, dotted line = interplate spaces. Scale bars: A-E = 0.02 mm.

The application of this histochemical test reveals strong positivity in the cytoplasm of the hepatocytes of the individuals (C/SD) as expected, indicating the presence of a great number of proteins. The nuclei of all cells (hepatocytes and Kupffer) also reacted strongly to the test (Figure 2A). The interplate spaces of the hepatic cells are negative to the test (Figure 2A).

##### Sedentary diabetic type 1 group (DB/SD)

No histochemical alterations were found in relation to C/SD except for the increase in the presence of erythrocytes, strongly stained in the light of the larger vases (Figure 2B).

##### Trained diabetic type 1 (DB/TR)

No histochemical alterations were found in relation to control group except for the increase in the presence of erythrocytes, strongly stained in the light of the larger vases (Figure 2C).

##### Sedentary diabetic type 1 and treated with aminoguanidine (DB/SD-AG)

No histochemical alterations were found in relation to control group except for the increase in the presence of erythrocytes, strongly stained in the light of the larger vases (Figure 2D). There is an increase in the space between the interplates, which are negative to the test.

##### Trained diabetic type 1 and treated with aminoguanidine (DB/TR-AG)

Concentration of positive protein material and erythrocytes between the hepatocytes plates is observed (Figure 2E).

#### Detection of neutral polysaccharides–PAS technique

##### Sedentary control group (C/SD)

This histochemical test reveals that the liver of the control individuals is positive for polysaccharides, which present strongly positive fine granulation distributed throughout the cytoplasm of the hepatocytes preferably located in the peripheral region of the cells (Figure 2F).

As this technique is not specific for the demonstration of the nucleus, Kupffer cells (previously identified in light microscopy by the nucleus observation) were not evidenced, and neither were the hepatocytes nuclei (Figure 2F).

##### Sedentary diabetic type 1 group (DB/SD)

The individuals from the sedentary group (DB/SD) present hepatocytes which react weakly to the test, or even do not react, demonstrating the reduction in the number of polysaccharides in the cytoplasm. Only in some regions of the hepatic parenchyma the presence of strongly positive rough granulation is observed. (Figure 2G).

##### Trained diabetic type 1 group (DB/TR)

The liver of the individuals subjected to the training presented an increase in the intensity of positive staining for polysaccharides with rougher granulation: concentrating preferably in some parts of the cell (periphery of the cells) (Figure 2H).

The application of this histochemical test also shows variation in the intensity of the reaction depending on the region of the organ. In the regions next to the centri-lobular veins weak positivity to the test is observed, and in farther ones strongly positive fine granulation is observed (Figure 2H).

##### Sedentary diabetic type 1 and treated with aminoguanidine (DB/SD-AG)

The hepatocytes of the individuals from this group show a similar reaction to the one observed in the sedentary diabetic group (DB/SD); i.e., weak cytoplasmic positivity and strongly positive rough granulation in some regions (Figure 2I).

##### Trained diabetic type 1 and treated with aminoguanidine (DB/TR-AG)

The liver of the trained and treated individuals (DB/TR-AG) present the same distribution of polysaccharides observed in the liver of individuals from DB/TR group: with positive polysaccharide granulations distributed in great number throughout the cytoplasm of the hepatocytes (Figure 2J).

The results obtained are represented in Table 1.

**Table 1:** **Results of the histochemical tests applied on the liver of rats subjected to the following situations: Sedentary Control (C/SD), Sedentary Diabetic (DB/SD), Trained Diabetic (DB/TR), Sedentary Diabetic Treated with Aminoguanidine (DB/SD-AG), Trained Diabetic Treated with Aminoguanidine (DB/SD-TR)**

Histochemical tests	C/SD				DB/SD				DB/TR				DB/SD-AG				DB/TR/AG			
	NH	NK	EI	LV	NH	NK	EI	LV	NH	NK	EI	LV	NH	NK	EI	LV	NH	NK	EI	LV
**Bromophenol blue**	**+**	**+++**	**+**	**+**	**+**	**+++**	**+**	**++**	**+**	**+++**	**++**	**++**	**+**	**+++**	**+**	**++**	**+**	**+++**	**++**	**++**
**PAS**	**-**	**-**	**-**	**-**	**-**	**-**	**-**	**-**	**-**	**-**	**-**	**-**	**-**	**-**	**-**	**-**	**-**	**-**	**-**	**-**

(**+++**) strongly positive, (**++**) mediumly positive, (**+**) weakly positive, (−) negative, (**NH**) hepatocyte nucleus, (**NK**) Kupffer cell nucleus, (**EI**) interplate space, (**LV**) vases lumen.

## Discussion

The hyperglycemia (high level of glucose in the blood), is a characteristic found in individuals who suffer from DM types 1 and 2, being considered the primary causing factor of the micro and macrovascular complications in these individuals. Such complications cause chronic degenerations such as: cardiopathy, nephropathy, retinopathy and neuropathy, which affect the life quality of the individuals [25]. Among the vias which lead to the vascular lesions associated to DM, the one of endogenous formation of advanced glycation products, also called (AGEs), has been currently considered the most important [26].

The AGEs are highly reactive and cause damage to the cells, once they modify the intracellular structures, due to the interaction of AGEs with the proteins of the extracellular matrix (modifying the cellular signalization) and also modify the blood proteins or lipids. Thus, the AGEs react with several compounds present in the organisms of the diabetic individuals, altering their chemical and functional capability, causing oxidative stress, morphofunctional alterations and increase in the inflammatory processes [25, 27, 28].

Pimagedine is a compound which prevents the formation of AGEs [14], and also reacts as an antioxidant agent preventing the formation of ROS (reactive oxygen species) and the lipid peroxidation in the cells and tissues [29].

It was observed in this study that in the individuals from the sedentary control group (C/SD), the liver showed a well-known and morphology and histology described in the literature on mammals [30–32]; i.e., hepatic parenchyma composed by hepatocytes plates which anastomose permeated by sinusoidal capillaries and Kupffer cells (hepatic macrophages).

Morphological alterations were found in the livers of sedentary diabetic individuals, (DB/SD) probably caused by diabetes, such as the presence of smaller hepatocytes, suggesting the decrease of the cellular hepatic metabolism, once the glucose available in the blood could not be absorbed by the cell due to the decrease or absence of insulin, corroborating Remédio [33], who studied Wistar diabetic rats.

In the diabetic individuals subjected to training (DB/TR), the hepatocytes and their nuclei underwent a process of hypertrophy which increased their size when compared with the individuals from the control group (C/SD) and sedentary diabetic (DB/SD). According to Rhodes et al. [34] and Teh et al. [35], the cellular hypertrophy (gradual increase in the cytoplasmic area of the hepatocytes) would occur due to several factors, among them the occurrence of the metabolic overload imposed to the cell, which probably occurred to the individuals in this study, justifying the swelling observed in the hepatocytes of the trained individuals’ livers. The same situation was also observed for the rats from the sedentary diabetic group treated with aminoguanidine (DB/SD-AG).

In the liver of the individuals from the trained diabetic type 1 treated with aminoguanidine (DB/TR-AG) the hypertrophy of the hepatic cells persisted and here the largest hepatocytes were observed, with an increase in the interplates space, suggesting a positive effect and somatization of the training and treatment with aminoguanidine in groups (DB/TR) and (DB/SD-AG), which lead to the most relevant alterations observed in relation to the sedentary control group.

In this study, the use of histochemical techniques permitted the observation of the distribution of proteins and polysaccharides in the liver of the individuals from all the groups studied. Thus, the presence of proteins in the liver of the individuals from the sedentary control group (C/SD) was significant and demonstrated by the strong reaction to Bromophenol blue in the cytoplasm of the hepatocytes and cellular nucleus. Among the rats from the diabetic groups, greater alterations were not observed; i.e., in all the groups a similar distribution to the one found in the livers from sedentary control group was detected (C/SD), corroborating data by Bond [36], on diabetic rats. However, the results oppose those by Bahnak and Gold [37] who reported that the synthesis of proteins in diabetic individuals would be reduced, due to the high protein catabolism (breakdown of protein to obtain energy in the absence of carbohydrate).

The polysaccharides, which play an important role in the hepatocytes once they are stored in form of glycogen, were also studied here. In the liver of individuals from the sedentary control group (C/SD), the polysaccharides were observed with strongly PAS positive fine granulation distributed throughout the cytoplasm. As the liver has the function to intermediate the conversion of energy from the food and supply extra-hepatic tissues, it is suggested that it would be capturing the glucose from the digestion and transforming it in glycogen to be stored in the hepatocyte [38].

In the sedentary diabetic group (DB/SD), a reduction in the staining for polysaccharides in most part of the hepatocytes’ cytoplasm was evident, corroborating Vallance-Owen [39]; Whitton and Hems [40], who also reported that the hyperglycemia in the diabetic individuals would be associated to the decrease of hepatic glycogen, due to a fault in the process of retaining glycogen in the liver. The lack of insulin and excess of glucagon, typical conditions of diabetic individuals, would affect the performance of the glycogenolytic and glycogenic enzymes, which would be contributing to lessen the storage of glycogen by the liver [41]. Thus, data obtained in this study suggest that in the diabetic individuals, the synthesis and the action of glycogenolytic and glycogenic enzymes would be impaired, which would decrease or prevent the synthesis of glycogen or increase in its breakdown (lysis) in the liver. This glycogen breakdown in diabetics would be a facilitator for the release of a greater number of polysaccharides to the blood, increasing the hyperglycemia and causing even more damage to the liver and the organism as a whole.

In the rats from the trained diabetic group (DB/TR), more intense staining for polysaccharides in the cytoplasm of hepatocytes was observed when compared with the individuals from the sedentary diabetic group DB/SD, suggesting that the regular and periodic physical activity would improve the process of hepatic glycogen accumulation (re-synthesis) [42, 43]. In the liver of trained individuals, probably by the lower production of glucose with consequent reduction of hyperglycemia.

Still in the liver of the individuals from this group a differentiated staining for polysaccharides was verified, varying according to the proximity to the larger vases (center-lobular veins). The hepatocytes located around them reacted weakly to PAS, suggesting that they would be the first to provide glycogen during the physical exercise, decreasing their reserve. Similar results were also found by Ferranini [44] in diabetic rats.

In the liver of the individuals from the sedentary diabetic group treated with aminoguanidine (DB/SD-AG), similar alterations to those from the sedentary diabetic group were observed (DB/SD): hepatocytes weakly positive to PAS indicating that the aminoguanidine did not interfere in the metabolism of polysaccharides which were stored in the liver of diabetic individuals.

In the trained and treated diabetic individuals (DB/TR-AG) polysaccharide staining and similar distribution to the one found in the liver of individuals from DB/TR group was detected, which would be justified by the interference of physical training and the absence of aminoguanidine (already confirmed in the previous group), confirming the benefits of moderate physical exercises for diabetic individuals, once there is an increase in the accumulation of hepatic glycogen. According to Guyton [8] the absence of insulin would impair the transportation of glucose to the interior of the cells, specially the muscucular, adipose and hepatic.

## Conclusions

The results obtained in this study suggest that the aminoguanidine is not hepatotoxic when used in the dosage of 1 g/L for the treatment of diabetes complications and also confirmed that moderate physical exercises reduced the damages caused by diabetes (increasing the utilization of glucose by muscular cells) without the use of insulin.

## Authors’ original submitted files for images

Below are the links to the authors’ original submitted files for images. Authors’ original file for figure 1 Authors’ original file for figure 2
