# Supplementary figures and images for: The action of aminoguanidine on the liver of trained diabetic rats
Source: J Diabetes Metab Disord. 2013 Jul 9;12:40. doi: 10.1186/2251-6581-12-40 (PMC7983740; doi:10.1186/2251-6581-12-40)

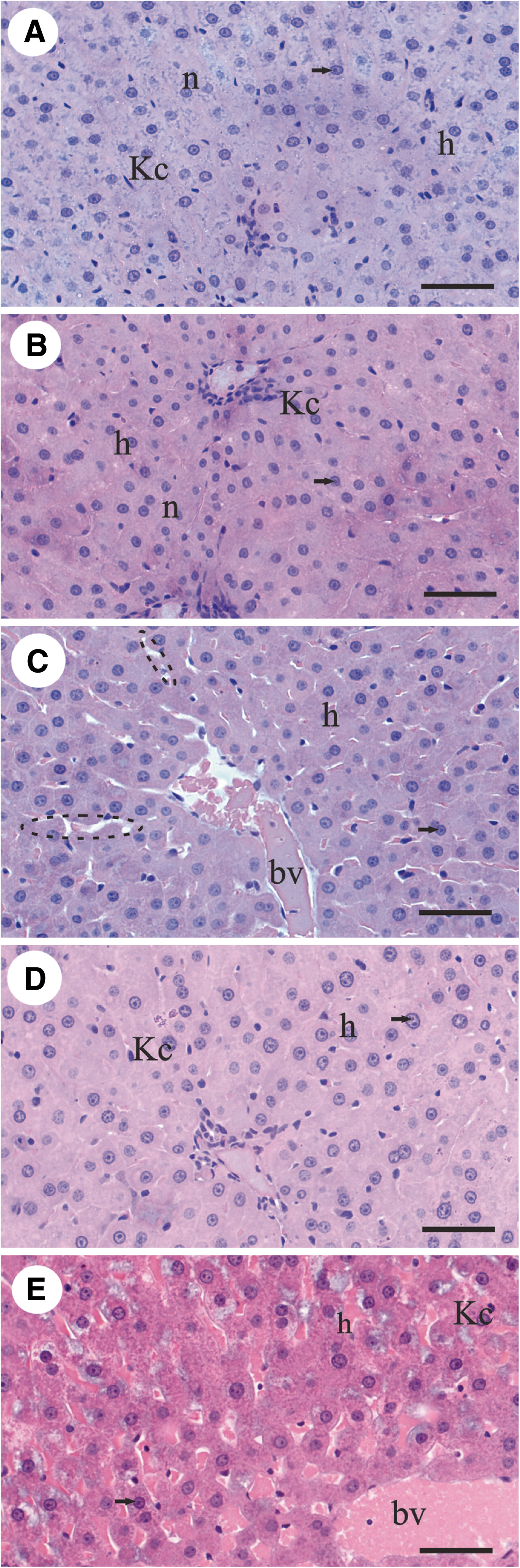

Supplement: Supplementary file 1 — Authors’ original file for figure 1 [file 40200_2012_64_MOESM1_ESM.tiff]

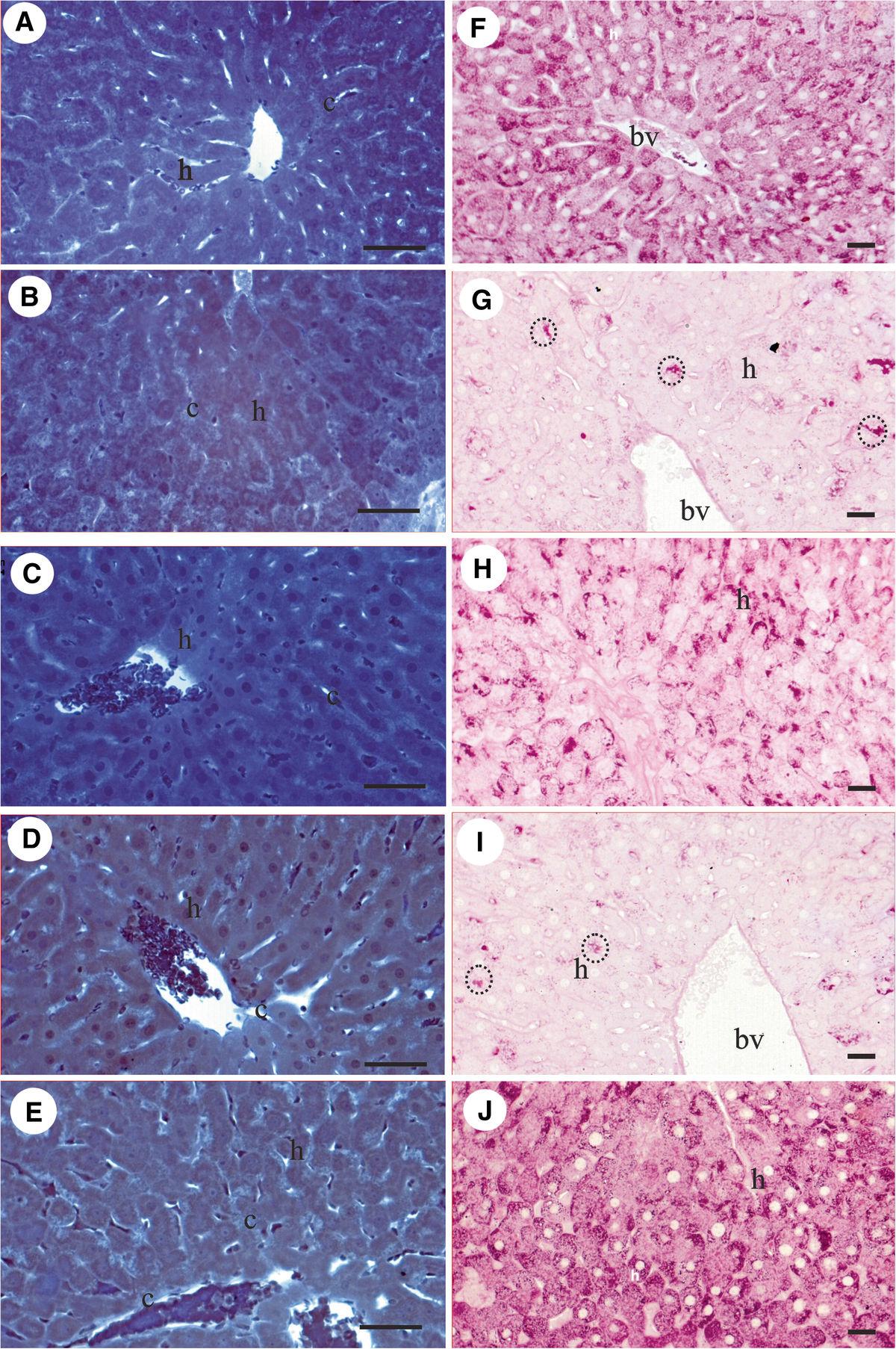

Supplement: Supplementary file 2 — Authors’ original file for figure 2 [file 40200_2012_64_MOESM2_ESM.jpeg]
